# CDK5RAP3, a New BRCA2 Partner That Regulates DNA Repair, Is Associated with Breast Cancer Survival

**DOI:** 10.3390/cancers14020353

**Published:** 2022-01-12

**Authors:** Jordi Minguillón, María José Ramírez, Llorenç Rovirosa, Pilar Bustamante-Madrid, Cristina Camps-Fajol, Gorka Ruiz de Garibay, Hermela Shimelis, Helena Montanuy, Roser Pujol, Gonzalo Hernandez, Massimo Bogliolo, Pau Castillo, Penny Soucy, Griselda Martrat, Antonio Gómez, Daniel Cuadras, María J. García, Javier Gayarre, CIMBA, Conxi Lázaro, Javier Benítez, Fergus J. Couch, Miquel Angel Pujana, Jordi Surrallés

**Affiliations:** 1 Genomic Instability Syndromes and DNA Repair Group and Join Research Unit on Genomic Medicine UAB-Sant Pau Biomedical Research Institute, Hospital de la Santa Creu i Sant Pau, 08041 Barcelona, Spain; MariaJose.Ramirez@uab.cat (M.J.R.); lrovirosa@carrerasresearch.org (L.R.); pbustamante@iib.uam.es (P.B.-M.); CCampsF@santpau.cat (C.C.-F.); hmontanuy@vhio.net (H.M.); MariaRoser.Pujol@uab.cat (R.P.); ghernandezv@uic.es (G.H.); massimo.bogliolo@uab.es (M.B.); pau.castillo@uab.cat (P.C.); 2 Center for Biomedical Network Research on Rare Diseases (CIBERER) U-745, 08041 Barcelona, Spain; 3 Program against Cancer Therapeutic Resistance (ProCURE), Breast Cancer and Systems Biology, Catalan Institute of Oncology (ICO), Bellvitge Institute for Biomedical Research (IDIBELL), L’Hospitalet del Llobregat, 08908 Barcelona, Spain; gderuiz@cnio.es (G.R.d.G.); griseldamartrat@gmail.com (G.M.); daniel.cuadras@sjd.es (D.C.); mapujana@iconcologia.net (M.A.P.); 4 Department of Laboratory Medicine and Pathology, Mayo Clinic, Rochester, MN 55901, USA; hshimelis@geisinger.edu (H.S.); couch.fergus@mayo.edu (F.J.C.); 5 Department of Health Sciences Research, Mayo Clinic, Rochester, MN 55901, USA; 6 Genomics Center, Centre Hospitalier Universitaire de Québec Research Center and Laval University, Quebec City, QC G1E 6W2, Canada; penny.soucy@crchul.ulaval.ca; 7 Cancer Epigenetics and Biology Program, Bellvitge Institute for Biomedical Research (IDIBELL), L’Hospitalet del Llobregat, 08908 Barcelona, Spain; antonio.gomez@vhir.org; 8 Human Cancer Genetics Program, Spanish National Cancer Research Centre (CNIO), 28029 Madrid, Spain; mjgarcia@cnio.es (M.J.G.); jnavarro507@hotmail.com (J.G.); javier.benitez@cnio.es (J.B.); 9 Hereditary Cancer Program, Catalan Institute of Oncology (ICO), Bellvitge Institute for Biomedical Research (IDIBELL), L’Hospitalet del Llobregat, 08908 Barcelona, Spain; clazaro@iconcologia.net

**Keywords:** BRCA2, breast cancer, CDK5RAP3, DNA repair, chemoresistance

## Abstract

**Simple Summary:**

*BRCA2* is an essential gene for DNA repair by homologous recombination and is often mutated in families at risk of breast and ovarian cancer. In this study we identified CDK5RAP3 tumor suppressor as a new BRCA2-interacting protein. CDK5RAP3 negatively regulates DNA repair of double-strand breaks, providing a new mechanism of DNA damage resistance. Consistently, gene expression data analysis showed CDK5RAP3 overexpression in breast cancer is associated with poorer survival. Finally, we identified common genetic variations in the *CDK5RAP3* locus as potentially associated with breast and ovarian cancer risk in a large cohort of *BRCA1* and *BRCA2* mutation carriers.

**Abstract:**

BRCA2 is essential for homologous recombination DNA repair. *BRCA2* mutations lead to genome instability and increased risk of breast and ovarian cancer. Similarly, mutations in BRCA2-interacting proteins are also known to modulate sensitivity to DNA damage agents and are established cancer risk factors. Here we identify the tumor suppressor CDK5RAP3 as a novel BRCA2 helical domain-interacting protein. CDK5RAP3 depletion induced DNA damage resistance, homologous recombination and single-strand annealing upregulation, and reduced spontaneous and DNA damage-induced genomic instability, suggesting that CDK5RAP3 negatively regulates double-strand break repair in the S-phase. Consistent with this cellular phenotype, analysis of transcriptomic data revealed an association between low *CDK5RAP3* tumor expression and poor survival of breast cancer patients. Finally, we identified common genetic variations in the *CDK5RAP3* locus as potentially associated with breast and ovarian cancer risk in *BRCA1* and *BRCA2* mutation carriers. Our results uncover CDK5RAP3 as a critical player in DNA repair and breast cancer outcomes.

## 1. Introduction

BRCA2, the breast cancer type 2 susceptibility gene product, is a master regulator of the DNA damage response pathway. BRCA2 is involved in DNA repair by homologous recombination (HR) and maintains genomic stability to counteract the mutational load due to double-strand breaks (DSBs) and interstrand crosslink (ICL) inducers [1,2]. BRCA2 is a 3418-amino-acid protein with multiple domains that interact with numerous functionally related proteins, such as RAD51, MDC1 and FANCD2 [3,4,5]. Monoallelic and biallelic mutations in *BRCA2* cause hereditary breast and ovarian cancer and Fanconi anaemia (FA), respectively [6,7,8,9]. Similar causative associations have been described for other genes of the FA/BRCA pathway, including *BRCA1*, *PALB2*, and *RAD51C* [10,11,12,13,14,15].

CDK5 regulatory subunit-associated protein 3 (CDK5RAP3, also known as C53, LZAP or IC53) is a tumor suppressor that activates p53, induces genotoxic-dependent apoptosis, inhibits the G2/M checkpoint via CHK1 and CDK1, and reduces NF-κB activity, cellular invasion, and tumor growth [16,17,18,19]. Low levels of CDK5RAP3 are associated with a subset of head and neck squamous cell carcinomas (HNSCCs) and hepatocellular carcinomas (HCCs) as well as with poor survival [18,20]. In this study, a screening for physical interactors of BRCA2 identified CDK5RAP3, which emerged as a critical regulator of DNA DSB repair that is associated with breast cancer outcomes.

## 2. Materials and Methods

### 2.1. Reagents and Antibodies

Mitomycin C (MMC, M0503), diepoxybutane (DEB, ref. 202533-16) 5-bromo-2-deoxyuridine (BrdU, B5002) and Hoechst 33258 (B2883) and IGEPAL (I8896) were from Sigma (Madrid, Spain). Ethidium monoazide bromide (EMA, e1374) and Sytox Green (S7020) were from Thermofischer Scientific (Barcelona, Spain). PARP inhibitor Olaparib (S1060) was from Selleckchem (Munich, Germany). Propidium iodide (P3566) and RNAse (12091021) were from Invitrogen (Barcelona, Spain). Colcemid was from Gibco (Barcelona, Spain). Antibodies used included: mouse monoclonal to CDK5RAP3 (ab57817), rabbit polyclonal to BRCA2 (ab123491), mouse monoclonal to RPA32 (ab2175), rabbit polyclonal to actin (ab1801), rabbit polyclonal to GAPDH (ab9485) and mouse monoclonal to vinculin (ab18058) were from Abcam (Cambridge, UK). Rabbit polyclonal to RAD51 (SC8349) was from Santa Cruz biotechnology (Heidelberg, Germany).

### 2.2. Cell lines and Plasmids

U2OS, HeLa, and stable cell lines U2OS-CherryCDK5RAP3, U2OS DR-GFP, U2OS SA-GFP, U2OS EJ5-GFP and HeLa SA-GFP were grown in DMEM (Biowest, cat. L0104, (Nuaillé, France) supplemented with 10% FBS (Biowest, cat. S181B) and plasmocin 0.1 mg/L (Invivogen, cod ant-mpt, Toulouse, France). I-SCEI-expressing plasmid pCBAS, empty vector (pCAGGS) and GFP-expressing plasmid NZE-GFP was kindly provided by Dr. Maria Jasin (Memorial Sloan Kettering Cancer Center, New York). SA-GFP (plasmid 41594) and EJ5-GFP (plasmid 44026) were from Addgene (Watertown, MA, USA. For pmCherry-CDK5RAP3 construct, CDK5RAP3 cDNA was amplified with primers flanking the Sal I restriction site and cloned into pmCherry-C1 vector (Clontech, Saint-Germain-en-Laye, France). For DNA and siRNA transfection, we used lipofectamine 2000 (cat. 11668) and lipofectamine RNAiMax (cat. 13778), respectively, from Invitrogen.

### 2.3. Western Blot

U2OS, HeLa or HEK cells were seeded on 6 well plates, 24 h later were treated with hydroxyurea, MMC or left untreated, and 24 h later cells were lyzed and SDS-PAGE and blotting with indicated antibodies performed as previously described [21]. The uncropped western blots are shown in Figure S5.

### 2.4. siRNA Transfection Oligonucleotides

Oligonucleotides were purchased from Sigma with the following sequences: Luciferase 5′-CGU ACG CGG AAU ACU UCG A-3′; CDK5RAP3-1 5′-GGC AGG AGA UUA UAG CUC U-3′; CDK5RAP3-2 5′-GGU UCG GAA UGU CAA CUA U-3′; CDK5RAP3-3 5′-CCC UGA CAC UGC UUG AAU A-3′; BRCA2 5′-GGA UUA UAC AUA UUU CGC A-3′.

### 2.5. Analysis of BRCA2 Interactions

Yeast two hybrid screens were carried out as previously described [22]. To confirm CDK5RAP3 interaction with BRCA2, HEK 293T cells expressing an empty vector or Flag-BRCA2 were treated with double thymidine block (10 mM) followed by a 9 h release to enrich S-phase cells, or left untreated. Cells were lysed and subjected to immunoprecipitation (IP) with anti-Flag M2 antibody. Immunoprecipitates and whole cell lysates were immunoblotted with CDK5RAP3, BRCA2 or actin antibodies. In other experiments, HEK 293T cells were left untreated or synchronized to S-phase by treatment with 2 mM hydroxyurea for 18 h. Cells were lysed and subjected to IP with anti-IgG or anti-BRCA2 antibody, followed by immunoblotting with CDK5RAP3, BRCA2, geminin or actin antibodies.

### 2.6. Laser Microirradiation Experiments

Laser microirradiation experiments were reported earlier [23]. Briefly, pmCherryCDK5RAP3-transfected U2OS cells were sorted twice (FacsJazz, Becton Dickinson, Madrid, Spain) for stable transfection. Cells were plated, stimulated with 10 µM BrdU for 24 h, and before microscope analysis pre-treated 10 min at 37 °C with 5 µg/mL Hoescht 33342 (Life Technologies, Barcelona, Spain). Images were taken with an Olympus Fluoview 1000 confocal microscope (Olympus, Barcelona, Spain). For laser microirradiation studies, cells were microirradiated with 5 s pulses of spots or lines with the 405 nm laser at full power and images were taken every 5 s for up to 5 min after localized DNA damage. For time course experiments, Cherry-CDK5RAP3 fluorescence was quantified from at least 30 cells and averaged.

### 2.7. Survival and Clonogenic Assays

Survival experiments were reported before [24]. Briefly, U2OS or HeLa cells were transfected twice with luciferase, BRCA2 or CDK5RAP3 siRNA at a final concentration of 20–40 nM in 6-multiwell plates. 24 h later, 1 × 10^5^ cells were seeded in duplicate in 6-multiwell plates (or 35 mm dish plates for irradiation) and left untreated for 24 h more. Cultures were then exposed to mitomycin C (MMC), olaparib or γ-irradiated at the indicated doses. Three to four days after the treatment, cells were rinsed with PBS, harvested by trypsinization and counted. Survival is reported as the percentage relative to untreated controls. For clonogenic assays, cells were plated and treated with MMC or γ-irradiated at the indicated doses, left to grow for 10–14 days, and Giemsa stained; then, colonies were finally counted.

### 2.8. In Vitro Flow Cytometric Micronuclei (FCM) Assay

FCM assay was reported previously [21]. Briefly, 250,000 cells/well were plated in 6-well plates. Cells were then treated with ICL inducer DEB (0.05 or 0.1 µg/mL) for 3 days. After treatment, trypsinized cells were centrifuged at 800 rpm for 8 min. Supernatant was removed and cells were resuspended by vortexing. 25 µL of EMA solution (0.125 mg/mL in PBS with 2% of FBS) was added to cell suspension. For the photo-activation step, plates were placed on ice under the visible light from a light bulb located 30 cm above cells for 20 min. After the photo-activation step (EMA covalently binds the DNA of cells with disrupted membrane integrity—dead/dying cells), plates were protected from light exposure for the remaining steps of the staining procedure. 100 µL of cold PBS with 2% FBS were added, and cells were centrifuged at 800 rpm for 8 min. Supernatant was discarded and cells were resuspended by vortexing. Cell suspension was kept in room temperature for 20 min before cell lysis. Cells were then lysed in a two-step procedure. 100 µL of lysis solution 1 (0.584 mg/mL NaCl, 1 mg/mL sodium citrate, 0.3 µL/mL IGEPAL, 1 mg/mL RNase A, 0.2 µM Sytox Green) were added. Samples were incubated at room temperature for 1 h. After the incubation, 100 µL of lysis solution 2 (85.6 mg/mL sacarose, 15 mg/mL citric acid and 0.2 µM Sytox Green) were added into the samples and immediately vortexed. Samples were incubated at room temperature for 30 min and were stored into a cold chamber overnight until samples were measured. Flow cytometry analysis was performed with a FACSCalibur (Becton Dickinson). At least 20,000 EMA negative-Sytox Green positive events (divided nuclei) were gated per sample. To exclude events that are not MN or nucleus from live cells, we used different gates for analysis that included forward scatter, side scatter, Sytox Green and EMA fluorescence (see Supplementary Figure S2A for details). Micronuclei (MN) frequency was expressed as the number of MN per thousand nuclei obtained in the G plot. Percentage of cells arrested in the G2/M phase was obtained in the H plot.

### 2.9. DNA Repair Assays

HR, SSA and NHEJ assays were performed essentially as described [25,26,27]. For the HR assay, the U2OS DR-GFP stable cell line was used. For SSA and NHEJ assays, U2OS and HeLa cell lines were transiently transfected with SA-GFP or EJ5-GFP plasmids, selected with puromycin, and clonal cell lines obtained from limited dilution. Stable cell lines were confirmed to have specific GFP fluorescence upon I-SCEI but not upon empty vector transfection. For DNA repair assays, I-SCEI-endonuclease-expressing vector, empty vector or GFP-expressing vector were transfected in U2OS or HeLa cell lines for DNA repair assays. Cells were transfected with NZE-GFP, pCBAS or pCAGGS for 16–24 h with the indicated plasmids with Lipofectamine 2000 (Invitrogen) and, 24 h, later collected and analyzed by cell cytometry.

### 2.10. Analysis of Publicly Available Cancer Transcriptomic Datasets

To analyze the effect of CDK5RAP3 expression on the prognostics of ovarian and breast cancer patients, we generated Kaplan–Meier survival curves of these cancers with low or high expression of CDK5RAP3 by using Kaplan–Meier Plotter (www.kmplot.com accessed on 1 October 2017) [28]. For TCGA analysis, pre-processed and normalized gene expression data were downloaded from the corresponding TCGA repository (http://tcga-data.nci.nih.gov/tcga/tcgaHome2.jsp (accessed on 3 July 2021)). Expression profiles were clustered using the PAM50 signature. The signatures were compiled from the corresponding publications and corresponded to up-regulated resistance-associated genes. The *CDK5RAP3* signature correlations were computed using the PCC and the average *Z*-score value per gene set. The profiles were clustered using the R software package cluster.

## 3. Results

### 3.1. CDK5RAP3 Interacts with BRCA2 and Localizes to DNA Damage

**Figure 1:**
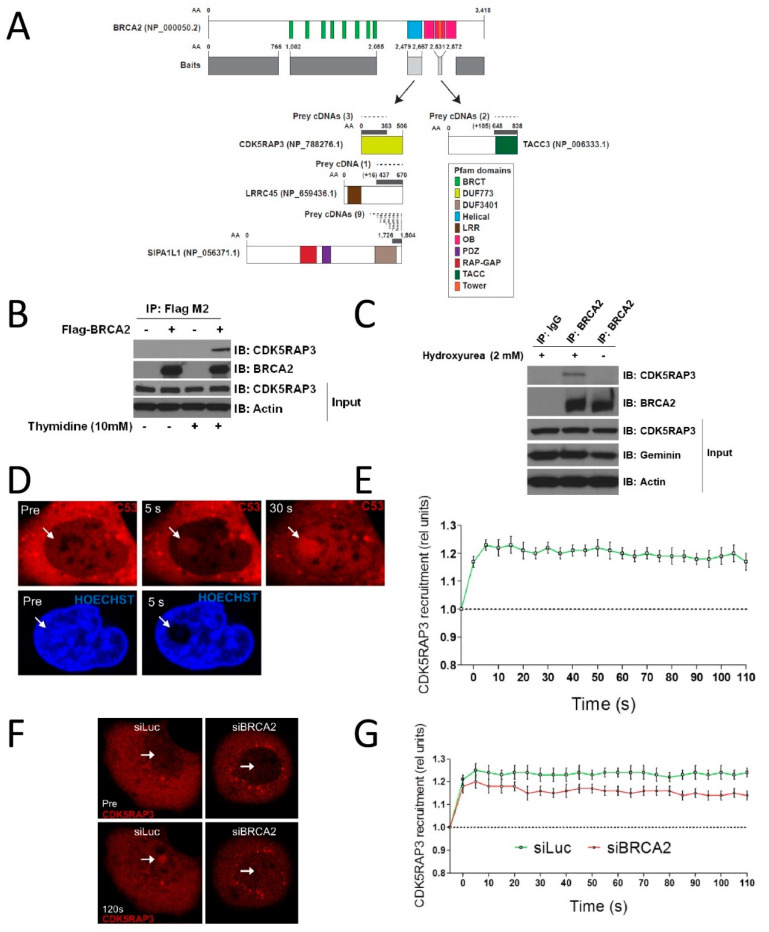
CDK5RAP3 interacts with BRCA2 and relocates to DNA damage. (**A**) BRCA2 fragments used as baits for Y2H assay and potential interactors found (see materials and methods). (**B**) BRCA2 immunoprecipitation in HEK 293T cells expressing empty vector or Flag-BRCA2, double thymidine treated to enrich S-phase cells, or left untreated. (**C**) Endogenous BRCA2 IP in HEK 293T cells, S-phase synchronized with hydroxyurea or left untreated. (**D**) Laser micro-irradiation of U2OS cells stably expressing Cherry-CDK5RAP3. Representative image shows very fast CDK5RAP3 localization to DNA damage sites. (**E**) Time course of CDK5RAP3 DNA damage localization. (**F**) CDK5RAP3 localization after micro-irradiation in U2OS cells upon BRCA2 depletion. Representative image shows reduced CDK5RAP3 relocation in BRCA2 depleted cells. (**G**) Time course of CDK5RAP3 relocation to DNA damage upon BRCA2 depletion.

We screened for BRCA2 interactors by applying the yeast two-hybrid system and five baits based on Pfam-predicted domains and PONDR-predicted disordered regions [22], which overall covered most of the functionally important BRCA2 domains/regions (Figure 1A). The helical domain baits identified two potential interactors: CDK5RAP3 and LRRC45. The tower domain baits identified two additional potential interactors: SIPA1L1 and TACC3. Of these, we focused on CDK5RAP3, owing to its previously reported role as a tumor suppressor in hepatocarcinoma [18]. The CDK5RAP3–BRCA2 interaction was confirmed by coimmunoprecipitation (co-IP) in HEK293T cells. Cells were transfected with Flag-tagged BRCA2 and lysates were immunoprecipitated with anti-Flag antibody. Untransfected cells were immunoprecipitated with anti-BRCA2 antibody. As seen in Figure 1B,C, CDK5RAP3 interacted with BRCA2 in cells overexpressing Flag-BRCA2, as well as with endogenous BRCA2, only when cells were synchronized in the S-phase with double thymidine block or hydroxyurea treatment. These findings identified CDK5RAP3 as a novel physical interactor with BRCA2 and suggested that the association occurs during the S-phase of the cell cycle.

Once the CDK5RAP3-BRCA2 interaction was confirmed, we checked whether CDK5RAP3 relocalized to the site of DSBs by laser microirradiation experiments and confocal microscopy. As seen in Figure 1D,E, CDK5RAP3 relocalized to DNA damage shortly after microirradiation, and nuclear distribution was also enhanced after DNA damage. Depletion of BRCA2 resulted in reduced relocation of CDK5RAP3 to DNA damage, suggesting that the role of CDK5RAP3 in DNA damage repair is functionally connected to BRCA2 (Figure 1F,G).

### 3.2. CDK5RAP3-Depleted Cells Are Resistant DNA Damage

**Figure 2:**
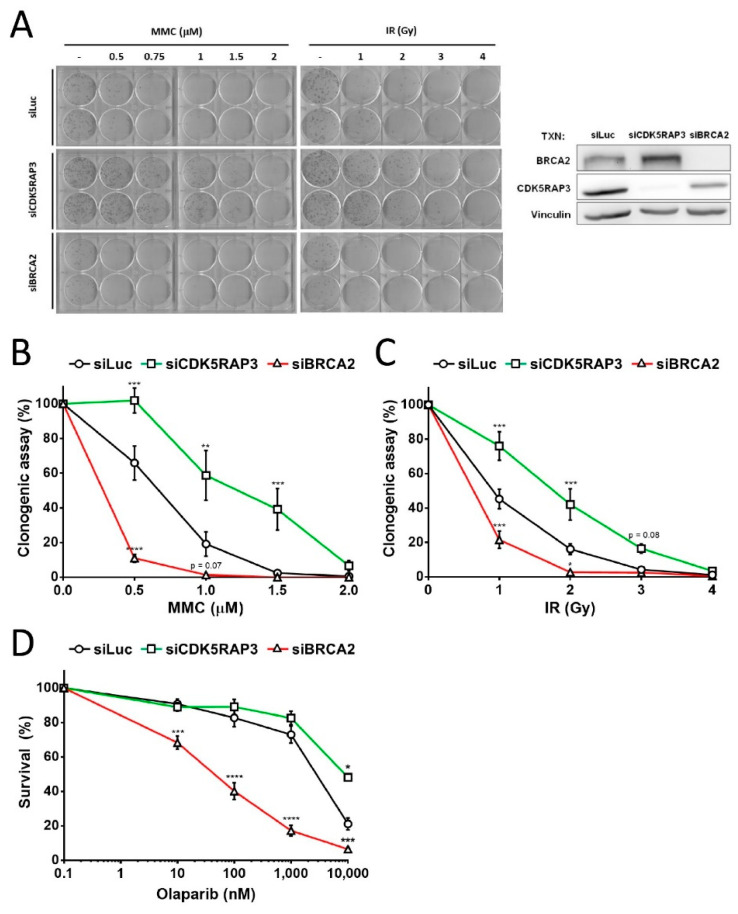
CDK5RAP3-depletion confers DNA damage resistance. (**A**) Clonogenic assay of U2OS cells transfected with siRNA targeting CDK5RAP3, BRCA2 or luciferase as a control (see materials and methods). Cells were plated and treated with different MMC (left) or ionizing radiation (right) doses for 10–14 days and Giemsa stained. Image shown is representative of at least three independent experiments with similar results. Right panel shows expression of siRNA targeted genes by Western blot. (**B**,**C**) Clonogenic assay graphs from averaged experiments done as in (**A**) in U2OS cells treated with MMC (**B**) or IR (**C**). (**D**) Survival assay of U2OS cells transfected as in (**A**), treated with different doses of PARP inhibitor olaparib. Graphs from (**B**,**C**) show mean ± SEM of at least 3 independent experiments. * *p* < 0.05, ** *p* < 0.01, *** *p* < 0.001, **** *p* < 0.0001.

It was previously reported that CDK5RAP3 affects sensitivity to DNA damage, as its inhibition reduced cell death and apoptosis in response to 20 μM etoposide and 2.5 Gy radiation treatment in HeLa cells [16]. Accordingly, we examined the effect of CDK5RAP3 depletion in the context of the HR pathway. In particular, we compared the DNA damage sensitivity of CDK5RAP3-depleted U2OS cells with that of BRCA2-depleted cells. Small interfering RNAs (siRNAs) were used to deplete CDK5RAP3 or BRCA2, and the expression reduction was confirmed by Western blot (Figure 2A, right panel and Figure S5). We then performed survival and clonogenic assays after treating the cells with increasing doses of an ICL inducer (MMC), a DSB inducer (ionizing radiation, IR), or a PARP inhibitor (olaparib). As expected, inhibition of BRCA2 rendered the cells highly sensitive to DNA-damaging agents (Figure 2A–D). However, CDK5RAP3-depleted cells became resistant to MMC (Figure 2A,B), IR (Figure 2A,C), and to a lesser extent, olaparib (Figure 2D). Similar results were seen in HeLa cells (data not shown). Finally, to discard the potential interference of cell growth in mutagen sensitivity, we analyzed cell cycle progression and proliferation, which were similar in CDK5RAP3-depleted cells in comparison with control cells (data not shown).

### 3.3. CDK5RAP3 Regulates DSB Repair and Genomic Instability

**Figure 3:**
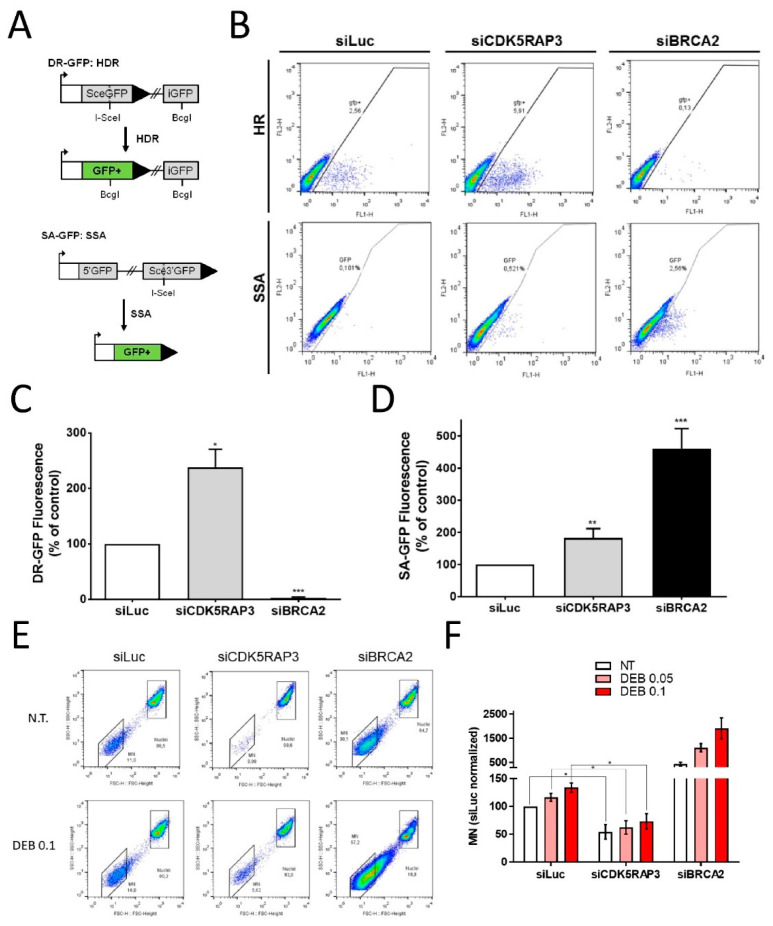
CDK5RAP3 regulates DSB DNA repair and genomic instability by homologous recombination and single-strand annealing. (**A**) Diagrams showing the in vitro homologous directed and single-strand annealing repair assays (see materials and methods). In the DR-GFP system (upper diagram), U2OS cells carry a plasmid inserted in the genome with two adjacent non-functional GFP coding fragments, one of them with an I-SceI restriction site. When a plasmid expressing I-SceI endonuclease is transfected, a specific DSB is generated in one GFP coding fragment, promoting recombination with the adjacent fragment and the formation of a full, functional GFP coding sequence resulting in fluorescent cells. In the SA-GFP system (lower diagram), U2OS or HeLa cells carry a plasmid inserted in the genome with two adjacent non-functional GFP coding fragments, one short 5′ fragment and a 3′ fragment, including one with an I-SceI restriction site. When a plasmid expressing I-SceI endonuclease is transfected, a specific DSB is generated in the 3′ GFP coding fragment, and a full, functional GFP coding sequence is formed after strand annealing, homolog searching and fusion with the 5′ GFP fragment. (**B**) Flow cytometry plots from DR-GFP (upper panels) and SA-GFP (bottom panels) in siRNA-transfected U2OS cells. (**C**) Averaged graph from at least four independent experiments of DR-GFP U2OS cells. GFP-transfected cells were used to normalize transfection efficiency. (**D**) Averaged graph from at least four independent experiments of SA-GFP in U2OS cells. (**E**,**F**) CDK5RAP3 inhibition reduces spontaneous and DNA damage-induced genomic instability. siRNA-transfected U2OS cells were plated and treated with ICL inducer DEB at different doses, and 72 h later samples were processed for the FCM assay and analyzed by cell cytometry (see materials and methods and Supplementary Figure S2A). (**E**) Representative experiment of the FCM assay in U2OS cells. Shown are FACS plots of nuclei and micronuclei (MN). (**F**) Averaged graph from at least four independent experiments performed as in (**E**) and normalized to luciferase control samples. Graph bars from (**C**,**D**,**F**) show mean ± SEM. * *p* < 0.05 ** *p* < 0.01 *** *p* <0.001.

Because BRCA2-deficient cells are HR defective [1,2], we examined whether CDK5RAP3 similarly regulates DSB repair. An in vitro HR assay was performed in U2OS cells by detecting the HR-dependent correction of an incomplete GFP expression cassette inserted in the genome (Figure 3A left panel) in the presence or absence of CDK5RAP3 [25]. Control cells with intact HR exhibited efficient functional GFP expression (Figure 3B, upper left panel). As expected, BRCA2 inhibition strongly abolished HR repair (Figure 3B upper right and Figure 3C). Interestingly, CDK5RAP3 downregulation resulted in a marked increase in the percentage of fluorescent cells (Figure 3B, upper middle and Figure 3C).

We previously noted in Western blots that BRCA2 expression was consistently increased when CDK5RAP3 was inhibited (Figure 2A, Figures S1A and S5). We wanted to know if other proteins involved in HR repair could have a similar expression profile upon CDK5RAP3 inhibition. As seen in Supplementary Figure S2B,C (and data not shown, uncropped images in Supplementary Figure S5), BRCA2, RAD51 and RPA2 expression increased in CDK5RAP3-depleted U2OS and HeLa cells, consistent with a global upregulation of HR repair.

It is well known that the lack of HR repair in BRCA2-deficient cells is overcome by increasing error-prone repair through homology-directed single-strand annealing (SSA) [26]. To determine whether CDK5RAP3 plays a role in SSA, we performed another in vitro cellular assay based on an SSA-dependent correction of an incomplete GFP expression cassette (Figure 3A left panel). Similar to the results of the HR assay, U2OS (Figure 3) and HeLa cells (not shown) with active SSA repair exhibited functional, although less active GFP expression (Figure 3B lower left panel). As anticipated, BRCA2 depletion resulted in a 5- to 30-fold increase in the number of fluorescent cells (Figure 3B lower right panel and 3D). Interestingly, CDK5RAP3 depletion also resulted in a 2- to 10-fold increase in the number of fluorescent cells (Figure 3B lower middle panel and 3D). To further assess the role of CDK5RAP3 in DSB repair, a third GFP reporter-based cellular assay for non-homologous end joining was performed in U2OS and HeLa cells [27]. This assay showed that depletion of CDK5RAP3 (or BRCA2) had little or no effect on non-homologous end joining (Supplementary Figure S1B–D and data not shown).

Genomic instability is a hallmark of BRCA2-deficiency upon treatment with chromosome-breaking agents, and frequently micronuclei (MN) production in cells is used to study chromosome fragility [29]. We used the in vitro flow cytometric MN (FCM) assay (see materials and methods) in the presence or absence of CDK5RAP3 [30]. Consistent with DNA damage resistance (Figure 2) and HR and SSA DSB repair induction upon CDK5RAP3 depletion (Figure 3A–D), U2OS and HeLa CDK5RAP3-depleted cells showed a marked reduction in spontaneous and DNA damaging-induced chromosome fragility (Figure 3E,F and data not shown).

### 3.4. CDK5RAP3 Expression Is Associated with Poor Breast and Ovarian Cancer Survival

**Figure 4:**
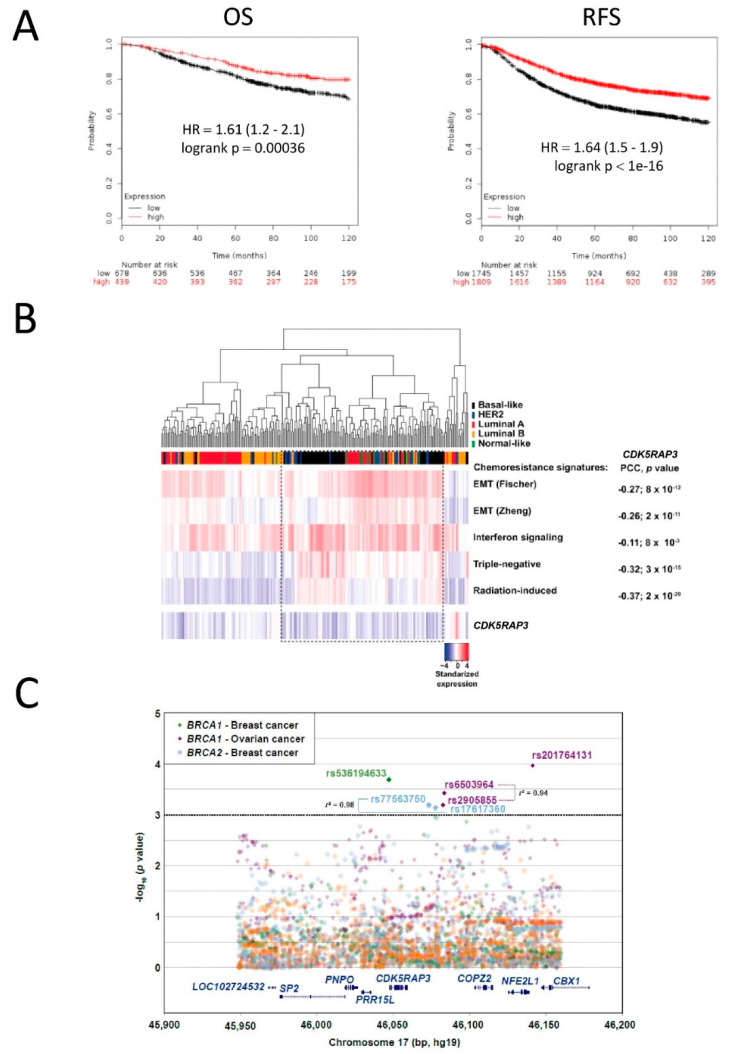
Low *CDK5RAP3* expression is associated with poor patient survival in breast cancer. (**A**) Kaplan–Meier survival curves for 1117 (overall survival, OS, left panel) and 3554 (relapse-free survival, RFS) patients with breast cancer. Data were divided at the best cutoff value into high and low expressing groups (for RFS, *n* = 1745 for CDK5RAP3 low, *n* = 1809 for CDK5RAP3 high; *p* < 10–16 with log-rank analysis; for OS, *n* = 678 for CDK5RAP3 low, *n* = 439 for CDK5RAP3 high; *p* = 0.00036 with log-rank analysis). Data obtained from the Kaplan–Meier Plotter (http://www.kmplot.com, accessed on 3 July 2021). (**B**) Unsupervised clustering of TCGA breast cancer gene expression data of CDK5RAP3 and defined signatures of chemoresistance and/or DNA damage resistance (two sets of epithelial-to-mesenchymal (EMT)-related genes are shown, as indicated in the text). The Pearson correlation coefficients (PCCs), corresponding *p* values, and tumor subtypes according to PAM50 classification are also shown. The dashed box highlights the cases with relative lower expression of CDK5RAP3 and higher for the signatures. (**C**) SNPs linked to CDK5RAP3 may influence breast/ovarian cancer risk in BRCA1/2 mutation carriers. Association plot for variants within the genomic region 100 kb on either side of CDK5RAP3 and risk of ovarian and breast cancer. X-axis position is referent to position (bp) on chromosome 17. Y-axis is −log10 (*p*-values) from association tests for risk of breast or ovarian cancer in BRCA1/2 mutation carriers, as shown in the inset.

Given that CDK5RAP3 interacts with BRCA2, whose mutations considerably increase the risk of breast and ovarian cancer, and that CDK5RAP3 downregulation leads to DNA damage resistance, we hypothesized that *CDK5RAP3* expression may be associated with poorer survival of breast and ovarian cancer patients. To test this hypothesis, we used the Kaplan–Meyer Plotter cancer transcriptomic database [28]. Data from 3,554 breast cancer patients revealed a significant association between low *CDK5RAP3* expression and poor overall survival (HR = 1.61; *p* = 3.6 × 10^−4^) and relapse-free survival (HR = 1.64; *p* < 10^−16^, Figure 4A,B). A similar but milder association was observed in patients with high-grade serous ovarian cancer (data not shown). Additional analysis also showed an association between poor survival rates and low *CDK5RAP3* expression in basal, luminal A and luminal B breast cancer patients (Supplementary Figure S3).

The above data show that lack of CDK5RAP3 leads to increased resistance to DNA-damaging agents in vitro and, in parallel, that low *CDK5RAP3* expression is associated with poor breast and ovarian cancer prognosis. To further assess the prediction that low *CDK5RAP3* levels promote tumor chemoresistance, we computed the expression correlation between *CDK5RAP3* and previously defined signatures of chemoresistance and/or DNA damage resistance using breast cancer data from The Cancer Genome Atlas [31]. The resistance signatures corresponded to sets of genes whose products were involved in epithelial-to-mesenchymal transition [32,33], induced by interferon signaling [34], by radiation [35], or associated with poor prognosis triple-negative breast cancer [36]. Remarkably, all analyzed signatures showed a significant negative correlation with *CDK5RAP3* (Figure 4B), specially for triple negative breast cancer; that is, low expression of *CDK5RAP3* was associated with relatively higher expression of the defined signatures, which, in turn, further endorses a functional link between CDK5RAP3 downregulation and chemoresistance.

### 3.5. SNPs in CDK5RAP3 May Be Associated with Cancer Risk in BRCA1/2 Mutation Carriers

Common genetic variation (represented by single nucleotide polymorphisms (SNPs)) at *loci* encoding for functional interactors of BRCA1 and/or BRCA2 modify breast and/or ovarian cancer risk in carriers of germline *BRCA1* and/or *BRCA2* mutations [37,38]. Since the *CDK5RAP3* gene product physically interacts with BRCA2 and functions in the DNA damage response, we conducted an association study using data from the Consortium of Investigators of Modifiers of *BRCA1/2*, CIMBA [39] (Figure 4C). Combined results of the iCOGS and Oncoarray studies identified potential associations with cancer risk in these settings (minor allele frequency (MAF) > 0.01, *p* values < 10^−3^, see supplementary results and methods): *BRCA1* mutation carriers, breast cancer risk, imputed rs536194633 HR = 0.90; and ovarian cancer risk, genotyped rs2905855 HR = 1.07, imputed rs201764131 HR = 0.91, and imputed rs6503964 HR = 0.93; *BRCA2* mutation carriers, breast cancer risk, imputed rs77563750 HR = 1.11 and rs17617360 HR = 0.90. Additional variants showed potential associations with ovarian cancer risk in *BRCA2* mutation carriers, but their MAFs were < 0.01 (imputation r^2^ values > 0.88; the complete locus results are shown in Table S1). Bioinformatic analysis of publicly available data suggested an enhancer role on genes at the corresponding genomic region by some of the depicted variants (Supplementary Figure S4, Table S2 and Supplementary Methods).

## 4. Discussion

Tumor formation and progression result from an imbalance in the expression of oncogenes and tumor suppressors. Oncogenes promote cell growth, and their overexpression or constitutive activation is highly deleterious. In contrast, tumor suppressors are essential for the regulation of uncontrolled cell division and maintenance of homeostasis, and normal cell functions may be compromised when they are downregulated or when their activity is impaired. DNA damage response pathways act as tumor suppressors by maintaining genomic stability and thus preventing oncogenic mutations. Similarly, cell cycle checkpoint pathways also act as tumor suppressors by controlling apoptosis and cell cycle progression, thereby allowing time for DNA repair.

CDK5RAP3 was first suggested to be a tumor suppressor when Wang and co-workers demonstrated functional interactions with p14^ARF^ and p53 [17]. Shortly thereafter, the same authors reported that CDK5RAP3 inhibits NF-κB signaling, providing additional evidence for its role in preventing tumor formation. Cells lacking CDK5RAP3 are more prone to cell invasion and exhibit enhanced xenograft tumor growth [18]. Interestingly, one-third of HNSCCs express remarkably low levels of CDK5RAP3, and low *CDK5RAP3* levels were associated with reduced survival in HCC patients [20].

In this study, we link for the first time CDK5RAP3 function with DNA repair by demonstrating that it physically interacts with BRCA2 and its absence leads to increased HR and SSA repair pathways. This is in contrast with BRCA2, whose depletion or mutation leads to HR abrogation compensated with induced SSA [26,40,41]. In addition, the observed DNA damage resistance and reduced genomic instability in CDK5RAP3-depleted cells may contribute to increased tumor aggressiveness and progression in breast cancer patients (as seen in Km plot analysis).

By yeast two hybrid experiments, we found that the BRCA2 helical domain interacted with CDK5RAP3 (Figure 1A), and this binding was further validated in co-IP experiments (Figure 1B,C). Some pathogenic missense variants have been identified in BRCA2 that map to the helical domain. They have been classified as pathogenic or not based on its capacity to disrupt HR pathway, but there are no reports on their role in SSA activity [4,42]. Thus, it would be interesting to address their capacity to maintain CDK5RAP3 interaction, in addition to SSA activity. HR induction by lack of CDK5RAP3 may be explained by BRCA2 expression induction (Figure 2A right and Supplementary Figure S2B,C), which could be produced through gene expression regulation at the transcriptional level or through protein stability mechanisms. However, how SSA activity increases in the absence of CDK5RAP3 remains elusive. Further experiments are needed to unravel the mechanisms by which CDK5RAP3 and BRCA2 regulate the HR and SSA pathways.

Our data connecting CDK5RAP3 to the BRCA2 pathway suggest a potential therapeutic approach for breast cancer. Blocking SSA activity is synthetically lethal in BRCA2-deficient tumors [43,44,45]. Similarly, because CDK5RAP3 deficiency could make tumors dependent on HR or SSA repair pathways, it is conceivable that inhibiting HR, SSA or both combined would also be synthetically lethal in tumor cells with low *CDK5RAP3* expression. Indeed, HR inhibitors targeting RAD51 and SSA inhibitors targeting RAD52 or XPF have recently been identified and are under development for clinical applications [46,47,48,49,50].

The functional connection between CDK5RAP3 and breast cancer may be extended to cancer etiology, as suggested by the results of the association study between common genetic variants and cancer development in *BRCA1/2* mutation carriers. The biochemical interaction with BRCA2 and the functional link to the DNA damage response support this hypothesis. In addition, functional annotation of the strongest risk variation indicates potential alterations of regulatory mechanisms. However, further genetic analyses combined with assays measuring the impact of specific variants are warranted in future studies.

In summary, in addition to the link to cancer risk, our study identifies *CDK5RAP3* expression in breast tumors as a new biomarker for poor prognosis and potential target for therapeutic intervention. Lack of CDK5RAP3 targets HR and SSA, two crucial tumor-suppressor DSB repair pathways, which can be added to its link with cell cycle arrest and p53 [17]. Interestingly, both BRCA2-deficient and p53-deficient mice have a carcinogenic phenotype independently. However, when both genes are simultaneously abrogated, tumor progression is extremely enhanced, and tumor cells acquire resistance to DNA-damaging drugs, a phenotype that resembles our findings related to CDK5RAP3 depletion [51,52,53,54]. CDK5RAP3 downregulation may therefore lead to the worst possible scenario for tumor progression because it confers resistance to DNA damage agents, reduces genome instability, and increases tumor aggressiveness.

## 5. Conclusions

CDK5RAP3 is a novel BRCA2-interacting protein that negatively regulates homologous recombination and single-strand annealing. CDK5RAP3 expression is linked to breast cancer survival and its genetic variations are associated with BRCA1/2 mutation carriers. CDK5RAP3 thus emerges as a potential prognostic biomarker for breast cancer.

## Data Availability

The data presented in this study are available in this article and supplementary materials.

## Supplementary Materials

The following are available online at https://www.mdpi.com/article/10.3390/cancers14020353/s1, Figure S1: CDK5RAP3 regulation in non homologous end joining. Figure S2: Genomic instability by flow cytometric micronucleus (FCM) assay and CDK5RAP3 role in HR-related protein expression. Figure S3: Low *CDK5RAP3* expression association with survival curves for different breast cancer subtypes. Figure S4: Functional annotation of the 17q21.32 region showing positions of candidate variants in relation to RefSeq annotated genes. Figure S5: Uncropped Western blots from Figure 2A (A), Supplementary Figure S1A (B) and Figure S2B (C), ladders are shown on the left side of the images, and cropped images marked as blue boxes. Table S1: Results of the combined iCOGS and Oncoarray studies at the CDK5RAP3 locus (imputed and genotyped variants) in BRCA1 and BRCA2 mutation carriers. Table S2: Functional annotation of potentially associated variants at the CDK5RAP3 locus.
