# Supplementary material for: CDK5RAP3, a New BRCA2 Partner That Regulates DNA Repair, Is Associated with Breast Cancer Survival
Source: Cancers (Basel). 2022 Jan 12;14(2):353. doi: 10.3390/cancers14020353 (PMC8773632; doi:10.3390/cancers14020353)
Supplement: Supplementary file 1 [file cancers-14-00353-s001.zip › cancers-1417863-supplementary.pdf]

cancers

Supplementary Materials: CDK5RAP3, a New BRCA2 Partner That Regulates DNA Repair, Is Associated with Breast Cancer Survival

Jordi Minguillón, María José Ramirez, Llorenç Rovirosa, Pilar Bustamante-Madrid, Cristina Camps-Fajol, Gorka Ruiz de Garibay, Hermela Shimelis, Helena Montanuy, Roser Pujol, Gonzalo Hernandez, Massimo Bogliolo, Pau Castillo, Penny Soucy, Griselda Martrat, Antonio Gómez, Daniel Cuadras, María J. García, Javier Gayarre, CIMBA, Conxi Lázaro, Javier Benítez, Fergus J. Couch, Miquel Angel Pujana and Jordi Surrallés

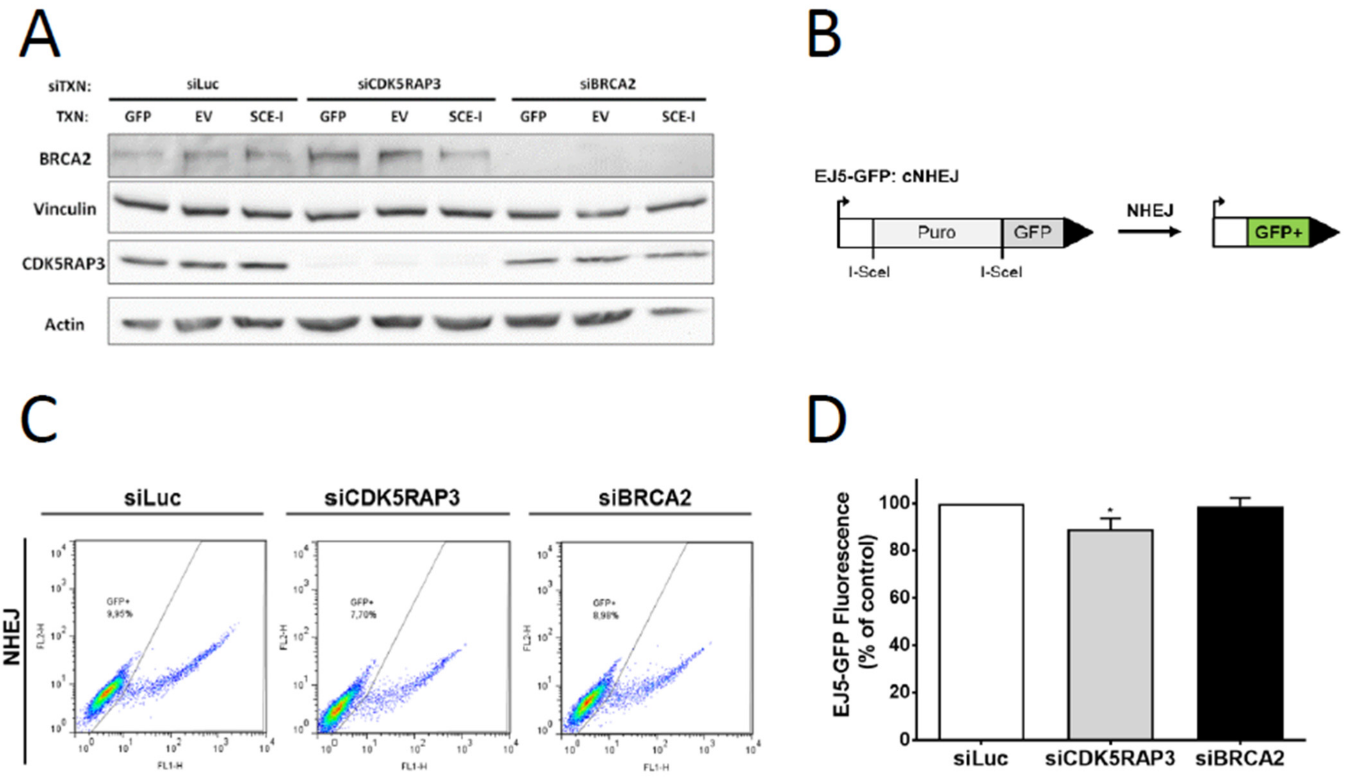

Figure S1. CDK5RAP3 regulation in non homologous end joining.

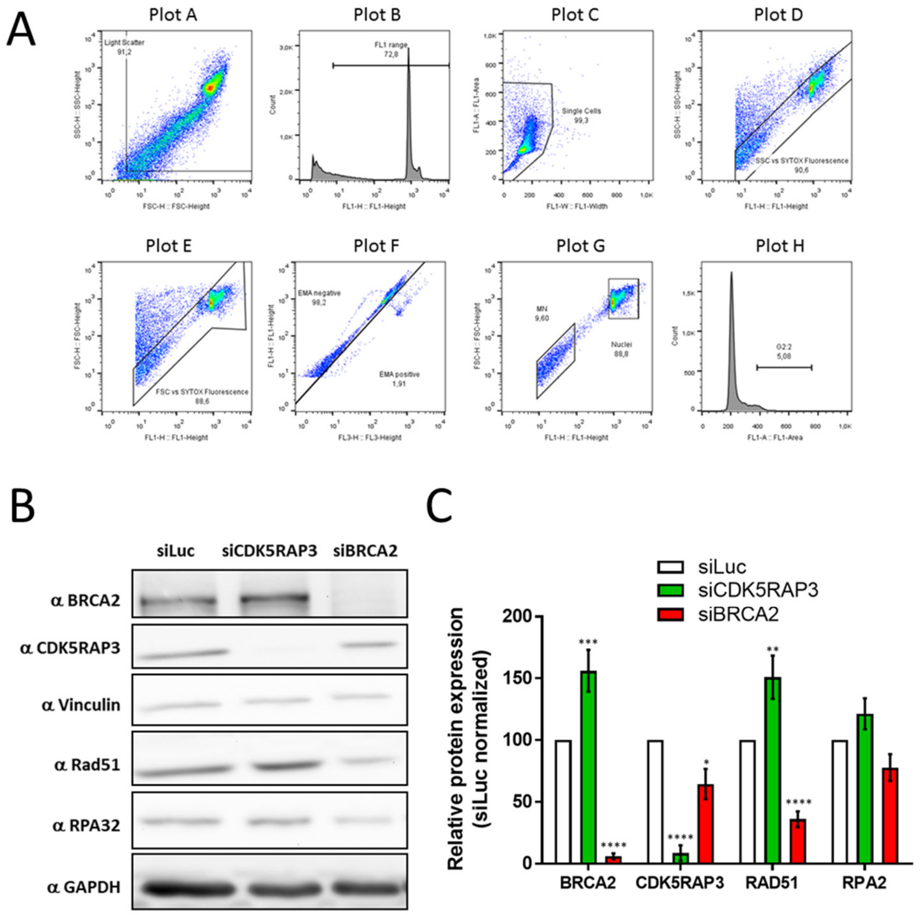

Figure S2. Genomic instability by flow cytometric micronucleus (FCM) assay and CDK5RAP3 role in HR-related protein expression. * $p < 0.05$, ** $p < 0.01$, *** $p < 0.001$, **** $p < 0.0001$.

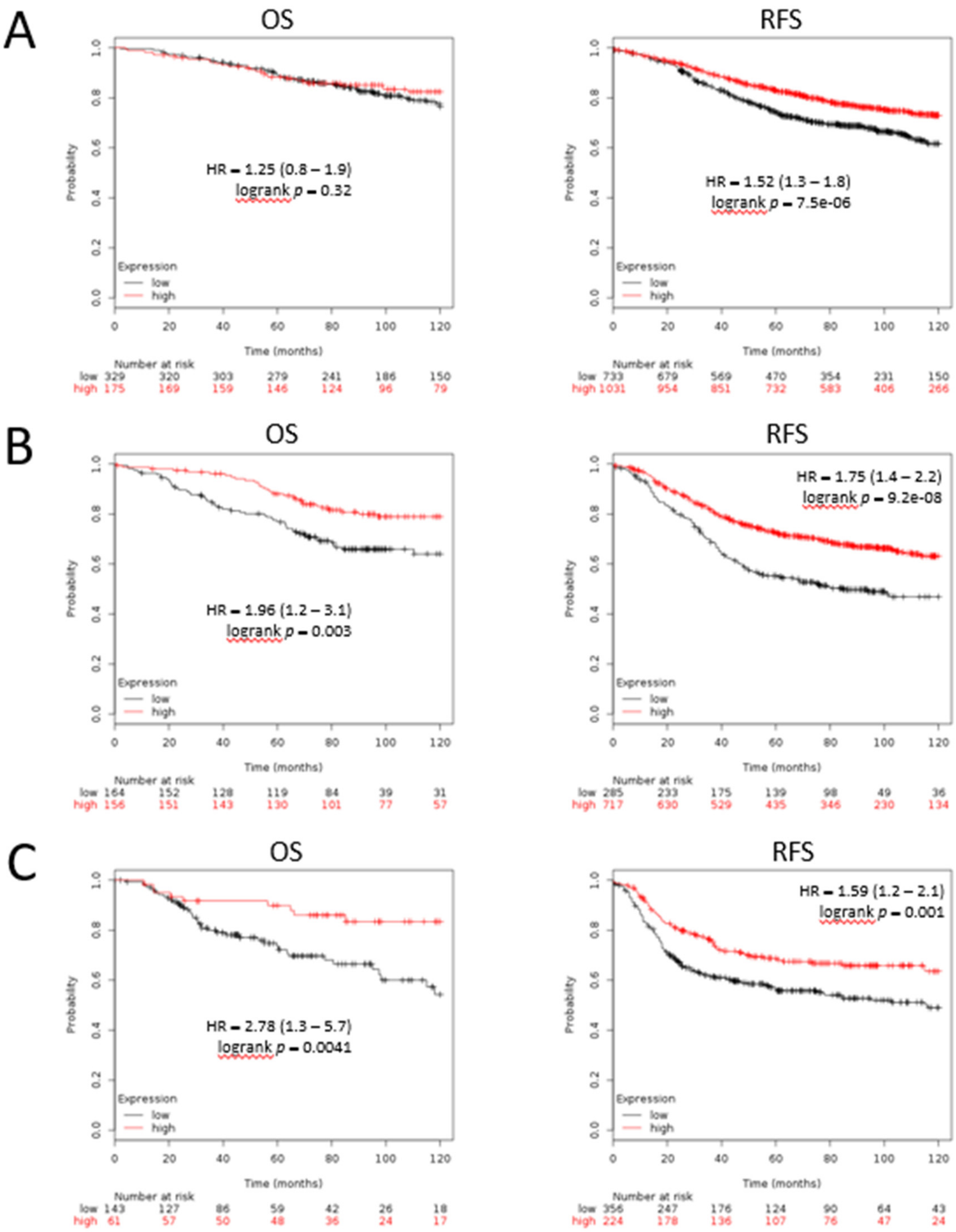

Figure S3. Low *CDK5RAP3* expression association with survival curves for different breast cancer subtypes.

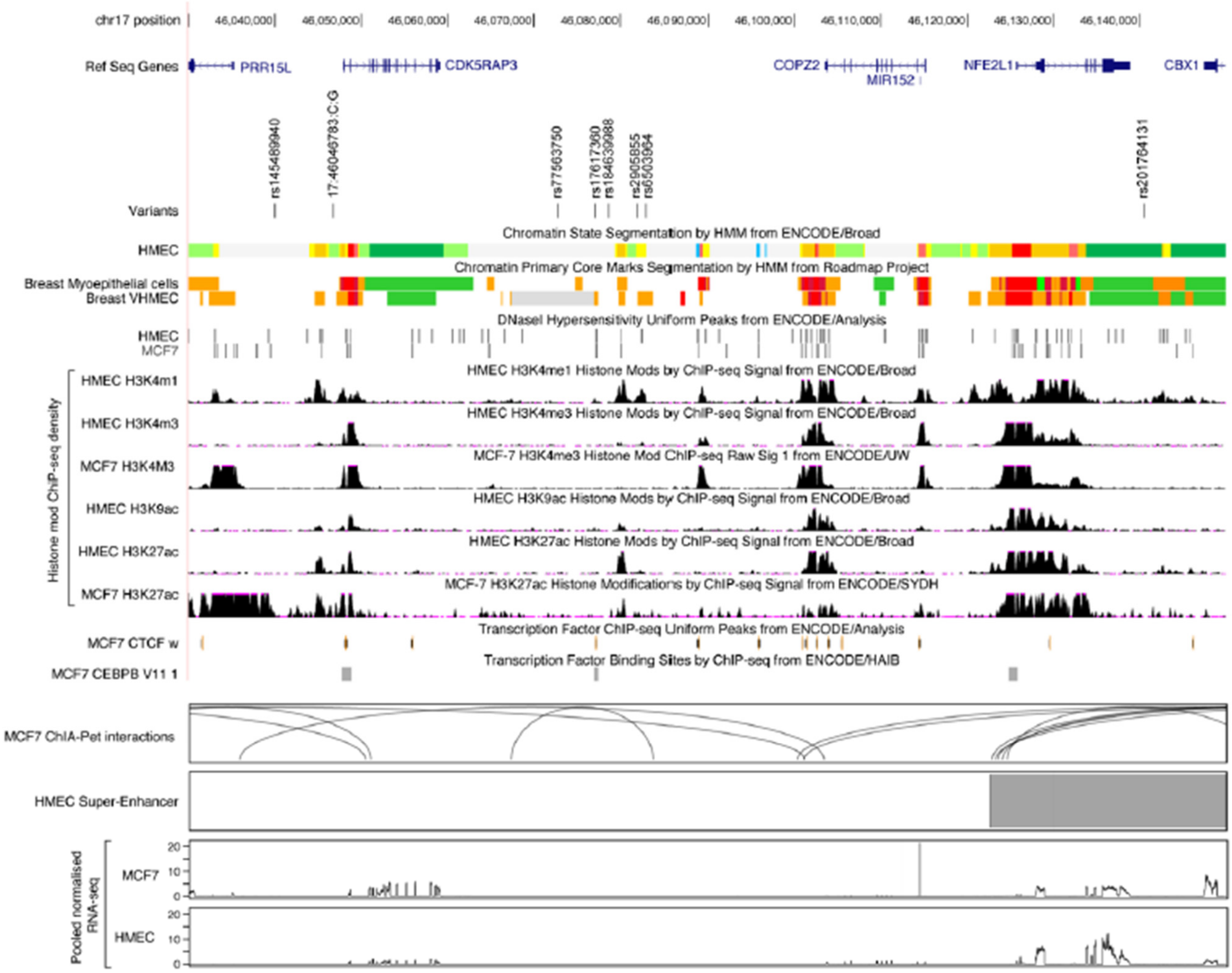

Figure S4. Functional annotation of the 17q21.32 region showing positions of candidate variants in relation to RefSeq annotated genes.

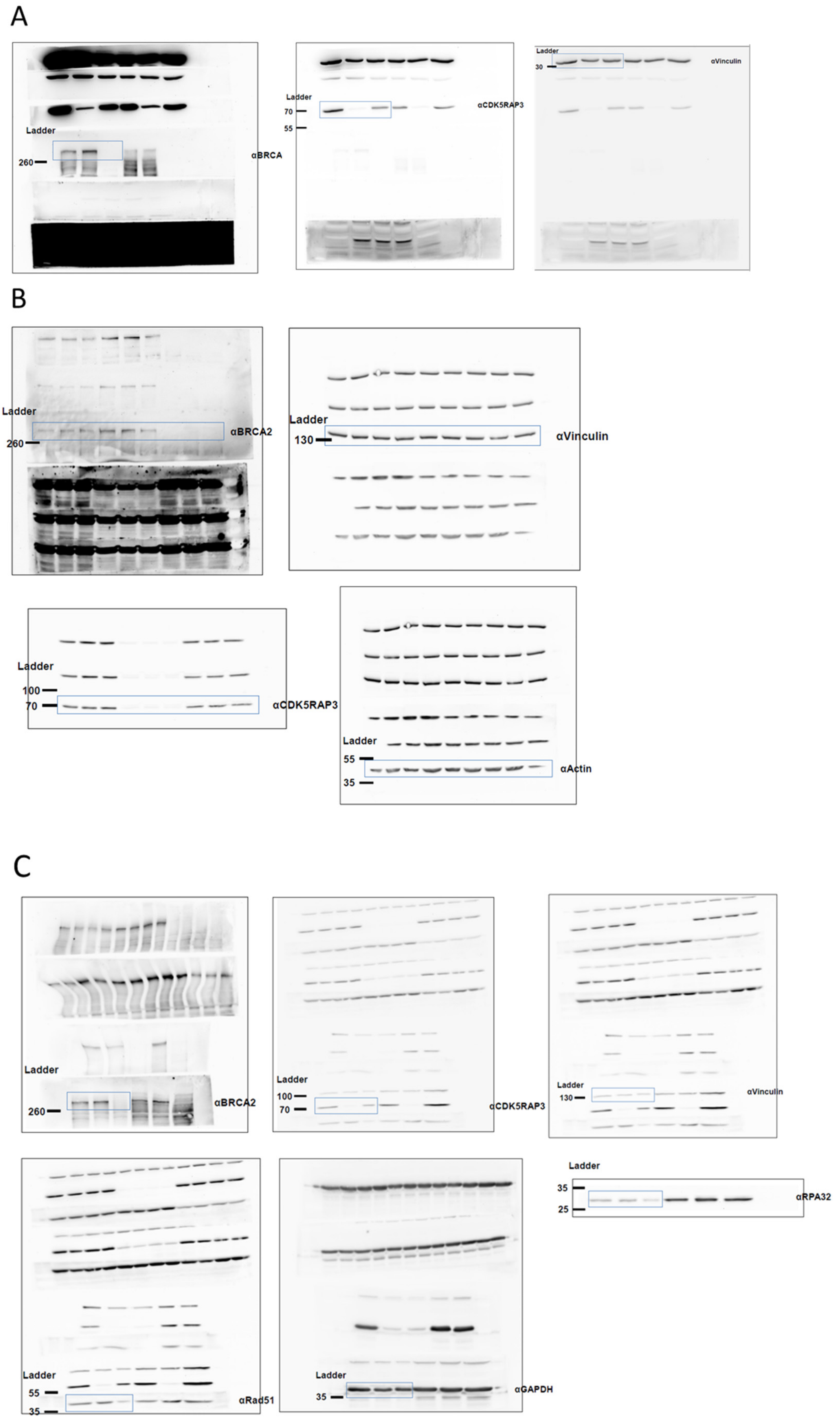

Figure S5. Uncropped Western blots from Figure 2A (**A**), Figure Suppl 1A (**B**) and Figure Suppl 2B (**C**), ladders are shown on the left side of the images, and original images marked as blue boxes.

1. Supplementary results

1.1. Putative function of CDK5RAP 3 variants associated with breast or ovarian cancer in BRCA1 and BRCA2 mutation carriers from the CIMBA consortium.

Functional annotation of the potentially associated variants with cancer risk in BRCA1/2 mutation carriers suggested relevant biological effects in breast cancer cells and/or normal mammary cell models (Figure S4 and Table S2). The variants rs77563750 and rs17673360 overlapped with H3K9Ac, a histone mark associated with promoters, and the latter variant also overlapped with a DNase hypersensitivity site and with a predicted weak enhancer. The variants rs2905855 and rs6503964 overlapped with H3K4me1, a histone mark associated with enhancers, and there were also predicted to lie in weak enhancers. In addition, analysis of enhancer-promoter links revealed interactions encompassing rs2905855 and rs6503964, as well as for rs145489940 targeting CDK5RAP3. Data on active mammary super-enhancers revealed an overlap between rs201764131 and such element [1]. Finally, analysis using RegulomeDB, a database that annotates SNPs with known and predicted regulatory elements, further supported two variants (rs77563750 and rs17617360) with high scores (2b), indicative of likely functionality. Variant rs77563750 may affect the binding of SMAD3, a transcriptional modulator activated by TGFβ, as determined by position weight matrix (PWM), while rs17617360 may affect the binding of CTCF as determined by ChIP-Seq (Table S2). While these predictions warrant further experimental assessments, this study identifies a novel molecular player of the DNA damage response potentially influencing breast and ovarian carcinogenesis.

2. Supplementary methods

2.1. CIMBA association study

Subjects included from the Consortium of Investigators of Modifiers of BRCA1/2 (CIMBA) were women of European ancestry aged 18 years or older with pathogenic mutations in BRCA1 and/or BRCA2. Most of the individuals were recruited through cancer genetics clinics and enrolled into national or regional studies. Sixty-seven centers from 23 countries contributed Oncoarray genotype data. After quality control, data were available from ~15,600 BRCA1 and ~11,000 BRCA2 mutation carriers. The details of the Oncoarray data quality control and analysis are provided elsewhere [2]. The Oncoarray association results were combined using fixed-effects meta-analysis with the association results from non-overlapping BRCA1 and BRCA2 samples genotyped using the iCOGS array [3,4] as described in Milne et al (in press). The combined results are shown in Table S1, and are based on ~18,860 BRCA1 mutation carriers (~9,520 breast cancer cases; ~2,900 ovarian cancer cases) and ~12,400 BRCA2 mutation carriers (~6,370 breast cancer cases, ~950 ovarian cancer cases). All mutation carriers provided written informed consent and participated under ethically approved protocols.

2.2. Functional annotation of variants

The following regulatory features were obtained for breast cell types from ENCODE and NIH Roadmap Epigenomics data through the UCSC Genome Browser: Chromatin Hidden Markov Modelling (ChromHMM) states, DNase I hypersensitivity sites, histone modifications of epigenetic markers more specifically commonly used marks associated with enhancers (H3K4Me1 and H3K27Ac) and promoters (H3K4Me3 and H3K9Ac), and transcription factor ChiP-seq data. To identify putative target genes, we examined potential functional chromatin interactions between distal and proximal regulatory transcription-factor binding sites and the promoters at the risk loci, using the Chromatin Interaction Analysis by Paired End Tag (ChIA-PET) dataset downloaded from 4D-genome [5]. Maps of active mammary super-enhancer regions in HMEC cells were obtained from Hnisz et al. [1]. Two publicly available tools, RegulomeDB [6] and HaploReg V4 [7], were also used to evaluate candidate variants. RNA-Seq data from ENCODE was used to evaluate the expression of exons across the 17q21.32 locus in HMEC and MCF7 cell lines. For HMEC and MCF7, alignment files from 4 and 19 expression datasets respectively were

downloaded from ENCODE using a rest API wrapper (ENCODExplorer) in the bam format and processed using GenomicAlignments and GenomicRanges R packages to normalize in Reads per Millions aligned, and to convert in coverages. The eQTL analysis results for each candidate variant and all genes within 1 Mb of it were looked up using the Genotype-Tissue Expression (GTEx) project portal in all available tissue samples [8].

Supplemental References

1. Hnisz, D.; Abraham, B.J.; Lee, T.I.; Lau, A.; Saint-André, V.; Sigova, A.A.; Hoke, HA.; Young, RA. Superenhancers in the control of cell identity and disease. *Cell* **2013**, *155,* 934–947.
2. Milne, R.L.; Antoniou, A,C. Modifiers of breast and ovarian cancer risks for BRCA1 and BRCA2 mutation carriers. *Endocr. - Relat. Cancer*. **2016**; *23*:T69–T84.
3. Couch, F.J.; Wang, X.; McGuffog, L.; Lee, A.; Olswold, C.; Kuchenbaecker, K.B.; Soucy, P.; Fredericksen, Z.; Barrowdale D.; Dennis, J.; et al. Genome-wide association study in BRCA1 mutation carriers identifies novel loci associated with breast and ovarian cancer risk. *PLoS Genet.* **2013**, *9*, e1003212.
4. Gaudet, M.M.; Kuchenbaecker, K.B.; Vijai, J.; Klein, R.J.; Kirchhoff, T.; McGuffog, L.; Barrowdale, D.; Dunning, AM.; Lee, A.; Dennis, J.; et al. Identification of a BRCA2-specific modifier locus at 6p24 related to breast cancer risk. *PLoS Genet.* **2013**, *9*, e1003173.
5. Teng, L., He, B., Wang, J., Tan, K. 4DGenome: a comprehensive database of chromatin interactions. *Bioinformatics* **2015**, *31*, 2560–2564.
6. Boyle, A.P.; Hong, E.L.; Hariharan, M.; Cheng, Y.; Schaub, M.A.; Kasowski, M.; Karczewski, KJ.; Park, J.; Hitz, BC.; Weng, S.; et al. Annotation of functional variation in personal genomes using RegulomeDB. *Genome Res.* **2012**, *22*, 1790–1797.
7. Ward, L.D.; Kellis, M. HaploReg: a resource for exploring chromatin states, conservation, and regulatory motif alterations within sets of genetically linked variants. *Nucleic Acids Res.* **2012**, *40*, D930–D934.
8. GTEx Consortium. Human genomics. The Genotype-Tissue Expression (GTEx) pilot analysis: multitissue gene regulation in humans. *Science* **2015**, *348*, 648–660.
